# Micronutrient status and associated factors of adiposity in primary school children with normal and high body fat in Colombo municipal area, Sri Lanka

**DOI:** 10.1186/s12887-020-02473-3

**Published:** 2021-01-06

**Authors:** Kalaichelvi Thillan, Pulani Lanerolle, Tharanga Thoradeniya, Dulani Samaranayake, Rohana Chandrajith, Pujitha Wickramasinghe

**Affiliations:** 1 Department of Biochemistry and Molecular Biology, Faculty of Medicine, University of Colombo, Colombo, Sri Lanka; 2 Department of Community Medicine, Faculty of Medicine, University of Colombo, Colombo, Sri Lanka; 3 Department of Geology, Faculty of Science, University of Peradeniya, Peradeniya, Sri Lanka; 4 Department of Paediatrics, Faculty of Medicine, University of Colombo, Kynsey Road, Colombo, Sri Lanka

**Keywords:** Micronutrient status, Adiposity, Sri Lankan children

## Abstract

**Background:**

The prevalence of obesity and associated risk of chronic diseases are increasing among the paediatric population. The effectiveness of preventive measures and interventions are likely to improve when all factors which associate with obesity in a specific target group are considered. Currently such comprehensive data is unavailable for Sri Lankan children aged 8–9 years.

**Methods:**

This paper pertains to the data collected from August-2015 to November-2016 for a case-control study which included cases (high body fat) (*N* = 160; males-81) and controls (normal body fat) (*N* = 164; males-80) recruited from primary schools in the Colombo Municipal area. Anthropometry and body composition (Bioelectrical impedance analysis-BIA) were measured. Diet, physical activity and socio-demographic data were collected using validated interviewer administered questionnaires. Serum concentrations of vitamins A, D [25(OH)D], E, folate (serum and red blood cell-RBC), zinc (Zn), selenium (Se), copper (Cu), iron (Fe), magnesium (Mg), calcium (Ca), chromium (Cr), manganese (Mn), cobalt (Co), ferritin, leptin and high sensitivity C-reactive protein (hs-CRP) were assessed using fasting blood samples.

**Results:**

Cases were from higher socio-economic strata and spent significantly less time on physical activities, more time on sedentary behaviours and consumed higher energy compared to the controls. Cases from both genders had significantly lower levels of vitamin D [25 (OH)D], Fe and Mg (*all p* < 0.05) and higher levels of Cu and Ca (*all p* < 0.01) compared to controls. Higher levels of ferritin and Cr were seen among male (*p* < 0.001) and female (*p* > 0.05) cases compared to the controls. However, total serum folate levels were lower in male (*p* < 0.01) and female (*p* > 0.05) cases while the RBC folate levels were higher among male (*p* < 0.01) and female (*p* > 0.05) cases compared with controls. Vitamins A, E, Se, Mn and Co (*p* > 0.05) were not significantly different between groups. The inflammatory markers, both hs-CRP and leptin levels were higher among cases (*p* < 0.001) compared to the controls.

**Conclusions:**

This study highlights higher socio-economic status, lower physical activity, more sedentary behaviours, higher energy intake and inconsistent distribution of micronutrients among the children with high body fat when compared with the control group. Increased levels of inflammatory markers indicate the presence of the risk of chronic inflammation in children with high body fat.

**Supplementary Information:**

The online version contains supplementary material available at 10.1186/s12887-020-02473-3.

## Background

Over the past few decades, the global prevalence of obesity has increased in epidemic proportion, especially among the paediatric population [1]. Based on the WHO’s recent estimates, the prevalence of overweight and obesity has increased from 4% in 1975 to 18% in 2016 among children and adolescents of 5–19 years, reaching over 340 million (https://www.who.int/news-room/fact-sheets/detail/obesity-and-overweight). This has led to the increase in prevalence of non-communicable diseases [2].

Changes in the dietary and physical activity patterns associated with economic development and urbanization known as the nutrition transition, observed in many low and middle income countries [3] has led to the epidemic of overweight and obesity. Obesity and associated chronic diseases are some adverse health outcomes of such nutrition transition [4]. Unhealthy dietary habits initiated during childhood, often continue throughout their life predisposing to chronic diseases in adulthood [5]. Socioeconomic factors such as education level and income of parents, [6] and number of siblings [7] are associated with the prevalence of overweight and obesity and are likely to work through dietary habits. The prevalence of overweight and obesity is increasing among adolescents and young adults in the South Asian region, including Sri Lanka as they experience the nutrient transition due to the rapid economic development and urbanization [8]. Further, overweight and obesity is a significant public health problem among adolescents in Colombo [9]. However, Sri Lankan children have been reported to have a higher percentage of fat mass from their younger age for a given Body Mass Index (BMI) [10].

Based on the epidemiological data, sex-specific differences were witnessed in the prevalence of childhood obesity [11]. The influence of an obesogenic environment on a child, as well as patterns of pubertal fat deposition differ in girls and boys. It is important to recognize the sex difference in micronutrient status and the inflammatory status associated with adiposity and expanding the study by stratifying on gender basis with the population specific body fat cut offs might provide more insights [12–14].

Although there is limited evidence on lifestyle parameters and overweight in children, such studies fail to provide a complete picture on the link between body fat and micronutrient status. Micronutrient deficiency is a major nutrition-related public health issue globally, particularly among children. Vitamin A, zinc, iron and folate deficiencies have been reported among children and adolescents in Sri Lanka [15, 16]. Since micronutrients play an essential role in energy metabolism, adequate intake of micronutrients is necessary for proper metabolism and tissue function [17] However, research into the links between micronutrient status and body fat have yielded conflicting results. Despite positive energy balance, evidence has directed attention towards micronutrient deficiency as a contributory factor to the deposition of fat and the pathogenesis of obesity [18]. Studies have reported a relationship between micronutrient status, mainly deficiencies among obese children [19]. On the other hand, micronutrient supplementation has also been shown to improve body weight in obese individuals [20]. While there is no evidence that explores the associations between micronutrients and body fat among Sri Lankan children, there is also no available data on the status of other important micronutrients such as selenium, copper, magnesium, chromium, manganese, cobalt and vitamin E in this population.

The role of chronic inflammation in obesity has been widely studied, and a more recent area of interest has been the possibility that low grade chronic inflammation may alter micronutrient status. It has been observed that inflammation reduces the vitamin and mineral levels irrespective of nutrient intake [21]. Hence, low grade chronic inflammation associated with high adiposity could be a contributory factor to micronutrient deficiency. Despite the increasing prevalence of overweight and obesity and concurrent micronutrient deficiencies among the child population in Sri Lanka, such a relationship has not yet been explored.

The risk of developing chronic disease among obese children varies between individuals and it is essential to identify potential risk factors beyond the conventional factors before planning preventive measures or interventions. Often, the failure of preventive measures is due to inadequate understanding of the complexities of the undelaying risk factors both biological and environmental. In developing comprehensive preventive strategies, an understanding of associated risk factors of obesity of a specific target group is a prerequisite.

Therefore, the present study aimed to compare micronutrient status, inflammatory markers, physical activity patterns, diet and socioeconomic factors in 8–9 year old children with high and normal body fat living in an urban area of Sri Lanka.

## Methods

### Study design, sample and subjects

This paper pertains to the data collected from August-2015 to November-2016 for a case-control study assessing the association between micronutrient status and adiposity carried out in primary school children aged 8–9 years in an urban area of Sri Lanka. The sample size was initially estimated based on the prevalence data of vitamin D and iron deficiency among two groups (obese/overweight and normal weight) of children based on published local and regional evidence [22–26]. The highest sample size required per group was 81, for an estimated prevalence of vitamin D deficiency of 47 and 25% among obese [23, 24] and normal weight [22] children respectively, an α error of 0.05, statistical power of 80% and considering a 10% dropout rate. Four groups were formed with the stratification by gender in both cases and controls. Further, the sample size adequacy was checked for the analysis of parameters of inflammation (with an α error of 0.05 and the power of 80%) using published mean values of leptin [27] among two groups of children.

Cases (*N* = 160; male − 81) were defined as those having body fat higher than the cut-off value (vide infra) and controls (*N* = 164; males-80) were defined as those with normal body fat.

### Subject recruitment

Thirty-seven schools were randomly selected from 110 schools within Colombo Municipal Council area. Overweight/obese and normal weight children were screened based on the BMI (overweight > + 1SD and < +2SD and obese ≥ + 2SD for each sex, based on BMI for age for 5–19-year-old children, WHO 2007 standards) during routine school medical inspections which are conducted by Medical Officers of Health of the area in all government and semi- government schools. The school medical inspection carried out among children in grades 1, 4 and 7, is a routine health screen, identifying problems for treatment or referral and includes the immunization programme. Children in grade 4 (aged 8–9 years) were considered for this study.

Selected children and their parents were invited with the help of their class teacher to the Clinical Laboratory of the Professorial Paediatric Unit of the Lady Ridgeway Hospital which is the main Children’s Hospital in Sri Lanka. After obtaining the written consent from parents, cases and controls were recruited according to their body fat measurements. Categorization of cases (male body fat > 28.6% and female body fat > 33.7%) and controls (male body fat ≤28.6% and female body fat ≤33.7%) were based on the percentage body fat cut-offs for Sri Lankan children [12].

### Inclusion and exclusion criteria

Eight to nine-year old children who were residents of the Colombo Municipal Council area and identified to have normal and high body fat by the cut off value (vide supra) were selected [12].

Children with any chronic illness and on long term medication, children on diet restrictions, birth defects, or congenital anomaly, children on micronutrient supplementation and children with a history of any allergies, infection occurring within 2 weeks of the study were excluded.

Ethical clearance for this study was obtained from the Ethics Review Committees of the Faculty of Medicine, University of Colombo (EC-14-168), and institutional approval from the Lady Ridgeway Children’s Hospital, Sri Lanka. Approval was also obtained from the Ministry of Education and the principals of schools to conduct the study. Informed written consent was obtained from parents/guardians of all participants.

### Anthropometric and body fat measurements

Height was measured to the nearest 0.1 cm without footwear using a potable stadiometer (SECA 225®; Germany), and weight was measured using an electronic weighing scale (SECA 803®; Germany) to the nearest 0.1 kg with light clothes. BMI was calculated as weight (in kilograms) divided by height squared (in meters). Waist circumference (WC) was measured at the midpoint between the lower border of palpable rib and iliac crest in the mid axillary line, using a non-stretchable tape to the nearest 0.1 cm and the waist-to-height ratio (WHtR) was calculated as waist circumference (in cm) divided by height (in cm). Body fat was measured by BIA technique using an In Body 230® multi-frequency analyzer (Biospace Co., Ltd., Seoul, Korea). All anthropometry measurements were performed according to WHO standards [28] using standardized equipment by a single investigator.

### Dietary intake

An interviewer-based food frequency questionnaire (FFQ) (attached as “Supplementary file 1”) was used to collect dietary information from the caregivers of the children.

#### Development of FFQ

The 106 food item FFQ was designed based on the questionnaire used in a previous study in Sri Lanka [29]. The food items were broadly categorized into pulses, rice and rice-based products, wheat flour-based products, breakfast cereals, bakery products, fast food, dairy products, fat/oil, nuts, vegetables/leafy vegetables, fish /meat and meat products/egg, fruits, confectionery, snacks and beverages. The consumption frequency of food item was assessed across the categories “how many times per day”, “how many times per week” and “No/rarely/1-2 times per month” with the portion size.

#### Validation of FFQ

The contents of the FFQ was evaluated by an expert panel of nutritionists from the Faculty of Medicine University of Colombo, Sri Lanka and the FFQ was modified based on their suggestions.

The validity of the FFQ was assessed against a 7-day food record in a convenient sample of the same study population. Spearman rank correlation coefficient was used to compare the frequency score for the food groups from the FFQ and the 7-day food record. The test-retest reliability (reproducibility) was assessed by administering the questionnaire to a sub-sample of the same study population on two occasions 1 week apart and by calculating the intra-class correlation coefficient (ICC) for frequency scores for the different food groups. The ICC values of < 0.4 and ≥ 0.76 were considered as poor and excellent reliability respectively, as reported in the previous study [30].

#### Assessing the dietary intake

The validated FFQ was administered to collect dietary information from caregivers by a single investigator. The questionnaire was administered with validated food portion size photographs [31] and commonly used household utensils including cups, bowls with measuring scales and serving spoons to estimate the portion sizes accurately. The amount (portion size in grams) and the frequency of consumption of each food item were assessed per week and total amount consumed per week was estimated by multiplying portion size by consumption frequency per week.

#### Estimation of nutrient intake

The nutrient and energy intake was calculated using the food composition tables of the United States Department of Agriculture (USDA) [32], Asian food composition tables [33] and locally published food composition tables [34–36]. The median nutrient intakes were compared with recommended dietary allowances (RDA) for Sri Lankan children aged 6–9 years [37]. Institute of Medicine (IOM) recommendations [38] were used when values were not available for Sri Lankan children. The daily intake of nutrients and energy was calculated as the average from a week’s intake.

### Identification and exclusion of under reporters of dietary intake

Under reporters of dietary intake were identified using the formula of Energy intake < Basal metabolic rate (BMR) × 1.2 which can be used as a cut off [39] where the BMR of each subject was calculated using Henry equation [40]. The food and nutrient intakes of children whose daily energy intake (Total energy intake per week/7) was below [BMR × 1.2], were excluded from the analysis.

### Physical activity

Physical activity was measured by a physical activity questionnaire (attached as “supplementary file 2”). The questionnaire was adapted from the children physical activity questionnaire (C-PAQ) (https://www.mrc-epid.cam.ac.uk/wp-content/uploads/2014/08/CPAQ.pdf) and modified to accommodate Sri Lankan traditions and culture. Some of the activities in the C-PAQ which were not relevant to Sri Lankan children were substituted with activities that are common. The modified questionnaire was validated using content and criterion validity and test-retest reliability (reproducibility) methods.

*Validation of the physical activity questionnaire.*

The contents of the physical activity questionnaire were validated, adopting a similar method used in validating the FFQ. The criterion assessment was carried out using ActiGraph wGT3x-BT® triaxial accelerometers https://www.actigraphcorp.com [41] in a different population of children of the same age. The children were instructed to wear the instrument on their right hip during their routine day to day activities except bathing, washing and water sports for seven consecutive days while maintaining a record of worn and removed time of the accelerometer every day. The child’s wearing time was defined as a minimum of 480 min or more on a day. The subjects who had completed at least two weekdays and 1 weekend day were included in the analysis. The average minutes of daily moderate to vigorous physical activity (MVPA) were computed [41]. Age-specific cut-off points were used to determine the minutes of MVPA [42]. The physical activity questionnaire was administered at the end of the week by the investigator. The total time spent on MVPA was calculated. The criterion validity was evaluated by assessing the significant difference between the measurements of mean time spent in MVPA by instrument and the time calculated by the questionnaire. The test-retest reliability was assessed by administering the questionnaire to a sub-sample of the same study population on two occasions one week apart with their daily routine activities. The intra-class correlation coefficient (ICC) was used to assess the test-retest reliability of the time spent on each activity, and it was considered acceptable if ICC > 0.7 [41, 43].

#### Assessment of physical activity

Physical activity was measured in a typical school week. This questionnaire covered areas related to physical and sedentary activities. The investigator completed the physical activity questionnaire by interviewing the caregivers on the frequency and the total minutes spent on each physical and sedentary activity by the child during the previous week. The activities were categorized based on metabolic equivalent (MET) values published in the Compendium of Physical Activity. Activities which had MET values of ≤1.5, 3–6 and > 6 were classified as sedentary behaviours [44], moderate and vigorous physical activity, respectively [45]. The time spent in both vigorous and moderate physical activities were summed to obtain the total time spent per week in moderate to vigorous physical activities (MVPA) and the average time spent per day was calculated.

### Laboratory analysis

A blood sample (8 mL) was drawn by an experienced nursing officer using stainless steel needles under standard sterile conditions after a 12 h overnight fast. Blood samples were collected into covered plain glass tubes without any additives as well as into an EDTA tube for RBC folate analysis. The blood sample collected in the glass tube was centrifuged at 3000 rpm for 10 min at 4 °C, and serum was separated, aliquoted and stored at -80 °C until the analyses were performed.

Serum Vitamin D concentration (25-Hydroxy vitamin D total) was determined using a competitive chemiluminescent immunoassay (Liaison® 25 OH Vitamin D Total, USA). Following an extraction method [46] serum vitamin A (*all-trans-retinol*) and vitamin E (α-tocopherol) concentrations were determined simultaneously at the wavelength of 292 nm by reverse phase (C18 column) HPLC (Shimadzu®, Japan) method with an ultraviolet (UV) detector using methanol and water (99:1 ratio) as the mobile phase with retinyl acetate as internal standard. Serum and RBC folate were determined using chemiluminescent assay (ARCHITECT-IP74®, USA) according to the manufacturer’s instructions.

Serum ferritin was determined using an enzyme link immunosorbent assay (ELISA) (AccuDiag™, USA) method according to the manufacturer’s instructions. Zn, Se, Ca, Mg, Cu, Fe, Cr, Mn and Co were determined using inductively coupled plasma mass spectrometry (ICP-MS) (Thermo ICapQ®, Fisher Scientific Inc., Bremen, Germany) after samples were digested with trace select nitric acid (≥69.0% Trace select; Fluka®, Germany), hydrochloric acid (≥37% Trace select; Fluka®, Germany) and hydrogen peroxide (35 wt% Sigma-Aldrich®, Germany) using microwave digestion system (CEM MARS 6®; USA) equipped with EasyPrep high pressure digestion vessels [47].

hs-CRP and leptin were assessed by a single step immunometric assay (Siemens Healthcare Diagnostics Inc., USA) and an ELISA (EIA-2395®, DRG International, Inc., USA) method, respectively.

### Quality control

All samples, where possible, were analyzed in duplicate, with random duplicate samples being analyzed when serum volumes were low. Low and high external quality control sera were used. Pooled serum was used as a bench quality control. The percent coefficient variance was maintained for each assay according to the standard protocol for each assay performed.

### Statistical analysis

Statistical analysis was performed using SPSS for windows version 20. The analysis was done separately for males and females. Normality of data distribution was assessed by the Kolmogorov-Smirnov test. The mean values of continuous variables were compared using independent sample t-test for variables which were normally distributed. Medians (interquartile range-IQR) were compared using non-parametric Mann –Whitney U test for the variables which were not normally distributed. The categorical variables were analyzed using chi-square test between cases and controls. Subjects with CRP > 10 mg/L were excluded in the comparison of CRP and ferritin between groups. The statistical significant level was set at *p* < 0.05.

## Results

Table 1 compares socio demographic, anthropometry and body composition data between case and controls. A higher percentage of cases were from families with a household earning of LKR ≥55,000 (USD ≥ 411.06) per month compared to controls in both sexes. A higher percentage of children in both control groups male and female, had more than one sibling compared to the cases. (The overall data on socio demographic, anthropometry and body composition of cases and controls without stratifying by sex were not shown and included in the Table S1 and attached as (“Additional file 1”).

**Table 1:** Socio demographic, anthropometry and body composition characteristics of cases and controls *N* = 324

Characteristics	Male			Female		
	Cases % BF > 28.6 *N* = 81	Controls %BF ≤ 28.6 *N* = 80	*p* value	Cases %BF > 33.7 *N* = 79	Controls %BF ≤ 33.7 *N* = 84	*p* value
Age in years	9.11 ± 0.323	9.21 ± 0.356	0.069^a*^	9.13 ± 0.266	9.11 ± 0.292	0.572^a**^
**Education level of father N (%)**						
Completed primary education	74 (91.4)	56 (70.0)	0.001^a*^	70 (88.6)	74 (88.1)	0.919^a**^
Not completed	7 (8.6)	24 (30.0)		9 (11.4)	10 (11.9)	
**Education level of mother N (%)**						
Completed primary education	75 (92.6)	59 (73.8)	0.001^a*^	64 (81.0)	74 (88.1)	0.210^a**^
Not completed	6 (7.4)	21 (26.2)		15 (19.0)	10 (11.9)	
**Employment status of parents N (%)**						
Both employed	18 (22.2)	8 (10.0)	0.035^a*^	31 (39.2)	21 (25.0)	0.051^a**^
One parent employed	63 (77.8)	72 (90.0)		48 (60.8)	63 (75.0)	
**Monthly income (LKR) N (%)**						
≥ 55,000.00	46 (56.8)	14 (17.5)	< 0.001^a*^	27 (34.2)	14 (16.7)	0.01^a**^
< 55,000.00	35 (43.2)	66 (82.5)		52 (65.8)	70 (83.3)	
**Number of siblings N (%)**						
≤ 1 sibling	36 (44.4)	19 (23.8)	0.006^a*^	33 (41.8)	29 (34.5)	0.341^a**^
> 1 siblings	45 (55.6)	61 (76.3)		46 (58.2)	55 (65.5)	
Height (m)	1.36 ± 0.06	1.31 ± 0.07	< 0.001^b*^	1.34 ± 0.05	1.29 ± 0.06	< 0.001^b**^
Weight (Kg)	39.46 ± 1.18	26.12 ± 1.22	< 0.001^b*^	37.90 ± 1.16	25.54 ± 1.19	< 0.001^b**^
BMI (kg/m^2^)	20.80 (19.1,23.2)	14.64 (13.8,17)	< 0.001^c*^	21.02 (19.2,22.3)	14.96 (13.7,16.3)	< 0.001^c**^
**BMI status N (%)**						
Normal	5 (6.2)	70 (87.5)	< 0.001^a*^	2 (2.5)	78 (92.9)	< 0.001^a**^
Overweight	51 (63.0)	10 (12.5)		57 (72.2)	6 (7.1)	
Obese	25 (30.8)	0 (00.0)		20 (25.3)	0 (00.0)	
%BF	35.8 (31.5,40.2)	16.26 (12.6,24.1)	< 0.001^c*^	38.4 (35.5,42.2)	20.7 (15.5,27.1)	< 0.001^c**^
BF (Kg)	13.8 (10.9,17.3)	3.85 (2.8,7.3)	< 0.001^c*^	14.6 (12.5,16.9)	4.95 (3.6,7.5)	< 0.001^c**^
WC (cm)	71.09 ± 1.08	55.37 ± 1.11	< 0.001^b*^	72.59 ± 1.12	54.82 ± 1.12	< 0.001^b**^
WHtR	0.53 ± 1.11	0.42 ± 1.09	< 0.001^b*^	0.53 ± 1.08	0.43 ± 1.09	< 0.001^b**^

*BMI* Body mass index, *WC* Waist circumference, *WHtR* Waist- to- height Ratio, *BF* Body fat

Monthly income categories were based on the mean household expenditure per month in Sri Lanka-. Household income and expenditure survey- 2016. LKR-Lankan rupee (1USD = 133.80 LKR on the day of data collection, 55,000.00 LKR = 411.06 USD)

^*^Differences between male cases and controls, ^**^Differences between female cases and controls, ^a^Pearson chi-square value, ^b^Independent sample t-test compared mean ± SD, ^c^Mann –Whitney U test compared median (inter quartile range)-statistically significant at *p* < 0.05

As expected, anthropometry and body composition measures were significantly higher in cases compared to the controls from both genders. The percentage of overweight and obese children assessed by BMI for their age and sex were 63 and 30.8% among male cases and 72.2 and 25.3% among female cases, respectively. Others had a normal BMI (−2SD − +1SD). Similarly, 87.5% of males and 92.9% of females from the controls group had normal BMI for their age and sex and rest were overweight (Table 1).

Table 2 compares the micronutrient status and inflammatory markers among cases and controls. Serum vitamin D [25 (OH)D] levels were significantly lower among the cases compared to controls. Similarly, the total serum folate levels were lower among cases than controls. However, the RBC folate levels were higher among cases compared to the controls. Serum vitamin A and E levels were not different between the two groups of both sexes.

**Table 2:** Micronutrient and inflammatory status of cases and controls *N* = 324

Characteristics	Gender	Cases Male-*N* = 81% BF > 28.6 Female-*N* = 79%BF > 33.7	Controls Male-*N* = 80% BF ≤28.6 Female-*N* = 84%BF ≤ 33.7	*p* value
Vitamin D (ng/mL)	Male	16.19 ± 5.06	17.72 ± 4.47	0.045^a*^
	Female	14.21 ± 4.13	17.09 ± 4.93	< 0.001^a**^
Vitamin A (μg/dL)	Male	61.94 ± 16.28	60.72 ± 17.62	0.647^a*^
	Female	63.13 ± 20.29	60.92 ± 17.41	0.461^a**^
Vitamin E (μg/mL)	Male	4.01 (2.9,5.2)	3.64 (2.9,5.1)	0.629^b*^
	Female	3.96 (2.9,5.4)	3.44 (2.2,4.7)	0.057^b**^
Ferritin (ng/mL)	Male	36.19 (23.6,46.7)	19.3 (8.4,29.5)	< 0.001^b*^
	Female	31.76 (21.6,40.5)	26.73 (16.7,39.5)	0.211^b**^
Total folate (serum)	Male	4.58 ± 1.43	5.36 ± 1.46	0.007^a*^
(ng/mL)	Female	4.72 ± 1.56	5.34 ± 1.44	0.055^a**^
Folate (RBC)	Male	239.01 (173.2,274.1)	189.29 (163.1, 236.9)	0.002^b*^
(ng/mL)	Female	234.39 (171.2,268.6)	208.91 (169.4,259.9)	0.303^b**^
Calcium (mg/dL)	Male	11.71 (10,12.8)	10.58 (8.7,11.8)	< 0.001^b*^
	Female	11.92 (8.6,12.7)	11.23 (7.4,12.2)	0.004^b**^
Magnesium (mg/dL)	Male	1.70 (1.6,1.9)	1.82 (1.7,1.9)	0.004^b*^
	Female	1.68 (1.5,1.8)	1.82 (1.7, 2.0)	< 0.001^b**^
Copper (μg/dL)	Male	142.77 (123.3, 159.7)	111.56 (90.5,135.9)	< 0.001^b*^
	Female	137.71 (102.5, 158.4)	112.87 (82.8,133.3)	< 0.001^b**^
Zinc (μg/dL)	Male	68.78 (57.7,83.3)	64.20 (51.4, 73.8)	0.025^b*^
	Female	63.02 (54.4,72.2)	65.71 (45.3,76.2)	0.797^b**^
Selenium (μg/dL)	Male	9.51 (8.4,10.2)	8.70 (7.2,10.5)	0.260^b*^
	Female	8.86 (6.9,10.2)	8.92 (7.0, 10.2)	0.784^b**^
Iron (μg/dL)	Male	61.93 (55.2,79.5)	90.84 (71.8,105.5)	< 0.001^b*^
	Female	70.24 (54.3,86.4)	94.88 (79.9,104.8)	< 0.001^b**^
Chromium (μg/dL)	Male	0.67 (0.32, 0.85)	0.21 (0.05,0.72)	< 0.001^b*^
	Female	0.54 (0.14, 0.71)	0.33 (0.11,0.74)	0.249^b**^
Manganese (μg/dL)	Male	0.28 (0.13, 0.61)	0.21 (0.09, 0.45)	0.187^b*^
	Female	0.20 (0.11, 0.37)	0.27 (0.09,0.67)	0.143^b**^
Cobalt (μg/dL)	Male	0.018 (0.004, 0.05)	0.02 (0.01,0.04)	0.551^b*^
	Female	0.013 (0.004, 0.03)	0.02 (0.01,0.04)	0.121^b**^
hs-CRP (mg/L)	Male	1.38 (0.4,3.4)	0.16 (0.08,0.5)	< 0.001^b*^
	Female	1.29 (0.6,2.6)	0.18 (0.05,1.03)	< 0.001^b**^
Leptin (ng/mL)	Male	9.84 (5.5,14.8)	2.19 (0.9,5.3)	< 0.001^b*^
	Female	15.09 (9.7,21.1)	3.71 (2.1,6.6)	< 0.001^b**^

*RBC* Red blood cell, *hs-CRP* High Sensitivity C-reactive protein, *BF* Body fat. The children with hs-CRP level > 10 mg/L excluded in ferritin and hs-CRP analysis, *Differences between male cases and controls, **Differences between female cases and controls, ^a^Independent sample t-test compared mean ± SD ^b^Mann-Whitney U test compared median (inter quartile range), statistically significant at *p* < 0.05

The serum concentration of Mg was significantly lower among male (*p* < 0.01) and female (*p* < 0.001) cases compared to controls. Although serum Fe levels were significantly lower in cases, serum ferritin levels were higher than in controls. Ca levels were significantly higher among both male (*p* < 0.001) and female (*p* < 0.01) cases compared to the controls. Similarly, Cu levels were higher among cases (*p* < 0.001) in both sexes and Cr among male (*p* < 0.05) cases compared to the controls but higher Zn levels were observed only in male cases compared to controls. There were no significant differences in the concentrations of Se, Mn and Co between groups in both sexes. Both CRP and leptin levels were significantly higher in cases (*p* < 0.001) compared to the controls in both male and female subjects.

Further analysis on the association of micronutrients and inflammatory markers (data not shown attached as an “Additional file 2”- Table S2), the median levels of hs-CRP and leptin were compared across the lowest and highest tertiles of micronutrients in the combined sample of both cases and controls. A significant reduction of hs-CRP and leptin levels was seen across the lowest and highest tertiles of vitamin D, folate, Mg and Fe while an increase was seen across the tertiles of ferritin and Cu. Similarly, a significant rise in hs-CRP and leptin was seen across the tertiles of Cr and Ca respectively. `.

### Dietary intake

Table 3 summarizes the median dietary intake of energy and other nutrients after excluding the under reporters (24 controls and 43 cases where the energy intake was <BMRx1.2). Overall, a higher proportion of under reporters was identified among males (*p* < 0.05) compared to females. Similarly, a higher percentage of under reporters was cases (*p* < 0.05) compared to controls. However, there was no significant difference in under-reporting between cases and controls within the gender groups.

**Table 3:** Daily median intake of nutrients of cases and controls *N* = 257

Nutrients intake/day	Gender	Cases M(*N* = 54)/F(*N* = 63)	Controls M(*N* = 63)/F(*N* = 77)	*p* value
Energy (Kcal)	Male	1908.19 (1614.7,2265.9)	1876.58 (1526.2, 2250.2)	0.299^*^
	Female	2109.10 (1805.9,2446.6)	1768.78 (1518.4, 2318.8)	0.003^**^
% Energy from CHO	Male	74.17 (70.5,76.7)	73.20 (68.3,75.9)	0.137^*^
	Female	74.92 (70.9,78.2)	72.74 (70.8,77.4)	0.491^**^
% Energy from protein	Male	12.15 (11.4,13.3)	12.31 (11.3,13.4)	0.588^*^
	Female	11.99 (11.0,13.0)	12.01 (11.1,13.4)	0.588^**^
% Energy from fat	Male	12.77 (10.5,16.1)	15.13 (10.3,17.8)	0.260^*^
	Female	12.61 (9.3,16.2)	13.63 (9.4,16.1)	0.392^**^
Vitamin A (μg RAE)	Male	918.33 (531.9,1432.3)	805.80 (580.9,1059.1)	0.394^*^
	Female	690.8 (418.3,993.1)	778.92 (484.2,1059.7)	0.447^**^
Vitamin D (IU)	Male	10.33 (6.3,19.1)	7.55 (3.7,15.8)	0.071^*^
	Female	10.56 (5.4,16.0)	9.50 (5.1,14.4)	0.253^**^
Vitamin E (mg)	Male	4.86 (3.2,7.5)	5.18 (3.0,7.0)	0.702^*^
	Female	4.24 (2.2,6.5)	3.56 (2.4,6.2)	0.620^**^
Folate (μg)	Male	72.04 (46.7,123.8)	104.23 (59.9,143.9)	0.064^*^
	Female	88.51 (54.5,126.7)	96.48 (47.5,142.4)	0.637^**^
Zinc (mg)	Male	5.40 (4.0,6.7)	5.26 (4.1,6.3)	0.983^*^
	Female	6.10 (4.9,7.6)	5.33 (4.0,6.9)	0.097^**^
Selenium (μg)	Male	50.65 (31.5,57.3)	42.28 (32.3,55.4)	0.163^*^
	Female	55.82 (41.1,73.3)	47.5 (36.0,61.0)	0.062^**^
Calcium (mg)	Male	432.25 (350.5,705.6)	525.81 (363.0,780.9)	0.372^*^
	Female	478.07 (232.7,763.4)	421.20 (293.7,629.2)	0.810^**^
Magnesium (mg)	Male	150.93 (107.2,225.7)	157.41 (99.8,251.1)	0.794^*^
	Female	156.06 (100.5,216.1)	148.73 (103.6,217.5)	0.870^**^
Iron (mg)	Male	14.64 (10.1,19.0)	15.36 (12.0,19.8)	0.415^*^
	Female	15.42 (12.9,18.2)	13.78 (11.4,19.0)	0.275^**^
Copper (μg)	Male	605.04 (412.6,840.0)	675.34 (415.9,1015.1)	0.455^*^
	Female	601.02 (411.7,932.2)	624.32 (410.7,980.1)	0.558^**^
Chromium (μg)	Male	29.72 (6.7,51.7)	21.59 (3.1,39.0)	0.035^*^
	Female	26.18 (7.8,41.2)	21.65 (6.8,41.6)	0.781^**^
Manganese (mg)	Male	2.54 (1.9,3.6)	2.34 (1.6,3.4)	0.424^*^
	Female	2.65 (2.0,3.4)	2.58 (1.7,3.3)	0.447^**^
Cobalt (μg)	Male	2.81 (1.9,3.3)	2.42 (1.7,3.1)	0.194^*^
	Female	2.61 (1.7,3.9)	3.27 (2.3,4.2)	0.059^**^

*CHO* Carbohydrate, *RAE* Retinol activity equivalent, *Kcal* Kilo calories, *IU* International unit

The total sample of *N* = 257 included in this analysis of dietary nutrient intake after excluding the under reporters

*Differences between male cases and controls, **Differences between female cases and controls, Mann-Whitney U test compared median (interquartile range)

The daily median energy intake of cases was higher compared to that of controls in both sex groups and significant among females. However, carbohydrate was the primary source of energy among all groups of children, and more than 72% of energy was from carbohydrate.

The daily median intake of micronutrients was not significantly different between cases and controls of this study population (Table 3).

The daily intake of vitamin D (RDA -600 IU), E (RDA-7 mg) and Folate (RDA-300 μg) in both groups were below the recommended levels for this age group (8–9 years) of children except for vitamin A (RDA-500 μg RAE), which was higher than the recommendation.

The intake of Se (RDA-21 mg/day), Mg (RDA-100 mg/day), Mn (RDA-1.5 mg/day), Cu (RDA-440 μg/day) and Cr (RDA-15 μg/day) were above the recommended levels for both groups while the median intake of Zn (RDA-6 mg/day), Ca (RDA-700 mg/day) and Fe (RDA-16 mg/day) were below the recommended level. However, assessment of Co was not possible as there was no RDA available.

### Physical activity

Cases spent less time per day on MVPA compared to the controls in both sex groups (Table 4). Among males, 28.4% of cases and 76.3% of controls met the recommendation (≥1 h/day) (*p* < 0.001) of MVPA whereas among females 44.3% of cases and 57.1% of controls met the recommendation.

**Table 4:** Physical and sedentary activity levels of cases and controls *N* = 324

Characteristics	Gender	Cases Males-*N* = 81%BF- > 28.6 Females-*N* = 79%BF > 33.7	Control Males-*N* = 80%BF- ≤ 28.6 Females-*N* = 84%BF- ≤ 33.7	*p* value
**MVPA**				
Total minutes spent/day	Male	37.14 (8.6,80.4)	95.86 (59.9,152.7)	< 0.001^a^
Median (IQR)	Female	50.0 (15.0,94.3)	72.86 (36.1,125.0)	0.005^a^
**Time spent N(%)**				
(≥1 h/day)	Male	23 (28.4)	61 (76.3)	< 0.001^b^
(< 1 h/day)		58 (71.6)	19 (23.7)	
(≥1 h/day)	Female	35 (44.3)	48 (57.1)	0.101^b^
(< 1 h/day)		44 (55.7)	36 (42.9)	
**Sedentary activities**				
Screen activities				
Total hours spent/week	Male	9 (4.0, 15.5)	7 (4,12)	0.074^a^
Median-IQR	Female	15 (5,18)	7 (4.2,12)	< 0.001^a^
Time spent N (%)				
≤ 14 h/week	Male	50 (61.7)	71 (88.8)	< 0.001^a^
> 14 h/week		31 (38.3)	9 (11.2)	
≤ 14 h/week	Female	31 (39.2)	72 (85.7)	< 0.001^a^
> 14 h/week		48 (60.8)	12 (14.3)	
Non- screen activities	Male	18.0 (13.3,26.6)	16.21 (11.3,20.6)	0.017^a^
Total hours spent/week (Median-IQR)	Female	17.92 (14,26.8)	15.21 (10.8,22.1)	0.017^a^

*MVPA* Moderate to vigorous physical activities, *Differences between male cases and controls, **Differences between female cases and controls, ^a^Mann-Whitney U test compared median (inter quartile range). ^b^Pearson chi-square, statistically significant at *p* < 0.05

Non screen activities included drawing, doing homework, listening to music, play cards, reading, siting and talking, travelling in vehicle

Categories of MVPA and screen activities were based on the physical activity and sedentary behaviour guidelines for Sri Lanka- 2018 Institute of Sports & Exercise Medicine, Ministry of Sports, Sri Lanka

On average, cases spent more time on screen devices compared to controls in both sexes. However, 61.7% of male and 39.2% female cases and 88.8% male and 85.7% female controls met the recommendation (≤14 h per week) of screen activities. Children in the high fat group spent significantly more time on non-screen sedentary activities than their counterparts with normal body fat.

## Discussion

To the best of our knowledge, this is the first study that compares micronutrient status, inflammatory markers, socio-economic factors, diet, physical activity and sedentary behavior patterns related to body fat among 8–9 year old school children in Sri Lanka. This study showed that higher socio-economic status, higher energy intake, lower physical activity, increased levels of inflammatory markers, reduced levels of serum vitamin D, folate, Fe, and Mg together with increased levels of RBC folate and serum Cu, Ca and Cr were associated with high body fat.

Higher body fat levels seen in children from a higher socio-economic sector is contrary to the pattern observed in developed countries where the prevalence of obesity was higher in low socio-economic sectors [48]. A similar trend has been reported among primary school children in China [49] and Sri Lankan adults [50] where obesity was seen among higher socio-economic strata. Apart from the fact that people with a high socio economic background have greater access to a variety of food, they may also have more access to computers and smartphones that would promote sedentary behaviour thus promoting weight gain. Further, less number of siblings were seen in the families of cases as reported in other studies [51]. Due to the lack of playmates, their choices of play is likely to be sedentary rather than physical activity.

Higher energy intake is one of the risk factors associated with weight gain. However, under- reporting is a significant problem in evaluating the energy intake in dietary assessments which may influence the estimation of energy intake [52]. A higher percentage of under- reporting has been reported in various populations compared to over-reporting. Further, under-reporting has been documented in relation to overweight [53]. Similarly, the prevalence of under-reporting was high among cases in the present study as well. Further, the present study identified a majority of under-reporters among males than females irrespective of body fat.

Thus in the present study, female cases (after excluding under reporters) were found to consume more energy compared to controls. However, the energy intake in all subjects exceeded the recommendation (males-1775 kcal/day, females-1725 kcal/day) [37] for this age group of children in Sri Lanka. Carbohydrate was the primary source of energy in this study population irrespective of body fat. As rice is the staple food in Sri Lanka, the frequency of consumption of rice and rice-based products is high. Besides (data not shown) a higher number of female cases had the habit of snacking mostly on sugared biscuits (≥2 times per day) and consuming sweetened carbonated beverages. All these unhealthy dietary habits may contribute to the high energy consumption among female cases.

Further, high sedentary behaviours, low physical activities [54] and higher energy intake of cases may probably associate with the risk of weight gain. On the other hand, a positive relationship has been reported between screen time and unhealthy eating behaviours among Polish adolescents [55]. However, the present study could not explain such relationship; instead, it showed a positive association between unhealthy eating behaviours and adiposity. Perhaps the screen time may directly or indirectly contribute to the risk of weight gain.

The increased levels of hs-CRP (> 1 mg/L) [56] and leptin in cases indicated the risk of inflammation. As leptin is secreted by adipose tissue, it increases in proportion to the fat mass. Leptin promotes inflammatory responses by inducing inflammatory cytokine production [57]. Inflammatory cytokines induce the production of CRP in the liver. Further leptin itself has been reported to induce the CRP production in cultured cells [58].

The low vitamin D levels (< 20 ng/mL) observed in both cases and controls is likely due to low dietary intake. The low dietary intake may probably be due to reduced dietary sources in Sri Lanka. The low vitamin D status of cases compared to the controls could be due to the inadequate sunlight exposure as they spent more time indoors with sedentary behaviours than physical activities. Moreover, volumetric dilution of vitamin D within the fat mass reduces its bioavailability as well [59].

Further, the low vitamin D status increases the parathyroid hormone (PTH) level [60]. Besides, a negative correlation between vitamin D and PTH as well as the lower mean levels of vitamin D in the high PTH group of Sri Lankan obese children have been reported [61]. Thus the increased PTH level could be the reason for the elevated levels of Ca among cases than controls. Another possible explanation would be the osteoclastic activity of increased levels of inflammatory cytokines in cases that may increase the Ca levels [62].

The investigators of previous studies have reported lower levels of vitamin A and E in obese children. Our findings demonstrated a marginal increase of vitamin A (retinol) and E (α-tocopherol) levels in cases than the controls. However, vitamin E intake was lower than the recommended level, while vitamin A was above the level in both groups. This probably could be due to the supplementation programmes as well as the emphasis on dietary vitamin A intake. Further, the increased levels of leptin seen in cases have been reported to induce oxidative stress [63]. Vitamins A and E are the fat-soluble antioxidants stored in the adipose tissue. The oxidative stress could be the possible cause for the release of these vitamins from adipose tissue to increase the blood level in cases.

In agreement with our study, low serum folate and increased RBC folate levels in obese individuals despite their low dietary intake of folate have been reported [64]. Serum folate reflects the recent dietary intake. The low serum folate levels in children with high body fat may be due to low dietary intake of folate or the volumetric dilution of folate within the blood. The low serum folate may stimulate the folate uptake by RBC in the intestine in cases as explained in a previous study [64].

Ferritin reflects Fe stores in the human body and is also an acute-phase protein. Obesity has been reported as an emerging risk of Fe deficiency [65]. The present study showed higher ferritin and lower Fe levels in cases compared to the controls. However, there was no significant difference in Fe intake observed between the two groups. A further potential explanation could be that; the low serum Fe level may be due to decreased Fe absorption by the action of hepcidin which has been reported to increase with inflammation associated with high adiposity [66].

A low level of Mg was observed in cases despite their higher dietary intake. While a mechanism cannot be suggested, it has been suggested in previous studies that it could be due to either decreased absorption or increased excretion of Mg in children with higher body fat [67].

In agreement with available data [68], the present study also showed higher levels of Cu in cases compared to the controls. Cu has been reported to play an essential role in the regulation of lipid metabolism [69]. Further, Cu regulates the uptake and utilization of primary metabolic fuels in the adipose tissue through the activity of semucarbazide-sensitive amine oxidase (SSAO). On the other hand, ceruloplasmin (Cp), a cuproprotein serves as a marker of inflammation. The higher levels of Cu in cases may be likely due to elevated levels these proteins as they require a continuous supply of Cu [70].

Contradictory to previous data [68] our data showed higher levels of Zn among male cases compared to controls but no difference was observed among females. There were no significant differences in dietary intake. Similar finding has been reported among obese children in Turkey [71].

Chromium is suggested to be a potentiator of insulin sensitivity [72]. A higher level of Cr was observed in cases than in controls. This could be due to higher dietary intake but the dietary intake was above the recommended level among cases as well as controls in both genders. The molecular mechanism for increased Cr level in high adiposity has not been elucidated. However, the documented higher consumption of carbohydrates mainly from rice and rice based products in Sri Lanka could be a contributory factor as rice has been reported to have higher Cr levels [73].

The present study showed no significant difference in Se levels between cases and controls, as reported in other studies [74]. Few studies have reported low Se levels among obese children compared to non-obese children [68]. Similarly, no difference was observed in Mn levels between groups. Higher dietary intake of Se and Mn among both cases and control could be the reason for the above findings of the present study. No significant differences were observed in serum Co levels between cases and controls, and we could not compare the serum levels and the dietary intake of Co as there is no recommended dietary intake.

Overall, the present study showed an inconsistent distribution of micronutrients in children with high body fat. Apart from the suggested mechanisms, inflammation could also play a role as it disturbs the micronutrient levels. However, we did not observe any significant change in inflammation in low and high levels of micronutrients among cases; instead, a significant difference was seen in the entire study population.

Although this study confines to a particular population in Sri Lanka with small sample size, it serves as one of the very few studies which described multiple factors associated with adiposity including lifestyle, dietary and biological factors (micronutrients and inflammation) in this age group of children at one glimpse. Further, sex-based description of data was another advantage as sex is an essential factor which influences the body composition as well as the other factors associated with adiposity. Therefore, the data of the present study could be served as preliminary data for future intervention based studies for all races in other countries.

### Limitations of the study

This study mainly focused on an urban paediatric population. This studied population was not a nationally representative sample. Therefore, it could not be generalizable to the entire Sri Lankan paediatric population of this age group. However, it could serve as a representative paediatric population of other urban areas of Sri Lanka where the similar socio-economic background is seen. Further, the sample size was calculated using the available prevalence data of two micronutrients, and the sample size may not have adequate power to detect the association of all micronutrients analyzed with adiposity. Moreover, population specific equations or gold standard method was not used for the identification of under reporters of energy intake. IOM recommended values were used since RDA values for the 8–9 year old Sri Lankan children were not available. However, the RDA values are given for 4–8 and 9-13 year old children and we used the RDA for 4–8 years for the comparison of dietary nutrient intake.

## Conclusions

Contrary to the findings reported in developed countries, the present study showed a significantly higher socioeconomic status among children with high body fat. Children with high body fat (cases) had lower physical activity, higher sedentary behaviours and higher energy intake compared to the controls. The children with high body fat had lower levels of vitamin D, total serum folate, Fe, Mg together with higher levels of RBC folate, Ca, Cu and Cr compared to the children with normal body fat. The increased levels of hs-CRP and leptin in children with high body fat indicate the risk of underlying chronic inflammation. Further, the significant changes in hs-CRP and leptin levels across the lower and higher levels of micronutrients may indicate the inconsistent distribution of micronutrients observed in the present study. However, future studies are recommended to assess the micronutrient status concerning the inflammation associated with high adiposity.

## Supplementary Information

**Additional file 1: Table S1.** Socio demographic, anthropometry and body composition characteristics of cases and controls (Sex specific and overall analysis).

**Additional file 2: Table S2.** hs-CRP and leptin levels across the tertiles of micronutrients in the total study population.

**Additional file 3: Supplementary file 1.** Food Frequency Questionnaire (FFQ). This FFQ included the food and beverage items commonly consumed by Sri Lankan children. The traditional food items included in this questionnaire were pittu, string hoppers, hoppers, rotti, koththu (made out of rice/wheat flour), fermented food item (made out of black gram and rice), kesel muwa (banana flower), brinjal (eggplant), ambarella (edible fruit with fibrous pit used for cooking) and kola kanda (porridge made out of green leaves, rice and coconut milk)

**Additional file 4: Supplementary file 2.** Physical activity questionnaire. This questionnaire was the adapted version of children physical activity questionnaire (C-PAQ) (https://www.mrc-epid.cam.ac.uk/wp-content/uploads/2014/08/CPAQ.pdf.) with culturally sensitive modifications where relevant.

## Data Availability

The data set used or analyzed during the current study is not publicly available due to the pending examination of the postgraduate student but are available with the corresponding author on a reasonable request.

## Abbreviations

RBC: Red blood cell
BMI: Body mass index
BIA: Bioelectrical impedance analysis
WC: Waist circumference
WHtR: Waist-to-height ratio
FFQ: Food frequency questionnaire
C-PAQ: Children physical activity questionnaire
USDA: United States Department of Agriculture
RDA: Recommended dietary allowance
IOM: Institute of Medicine
BMR: Basal metabolic rate
MET: Metabolic equivalent
MVPA: Moderate to vigorous physical activities
EDTA: Ethylenediamine tetraacetic acid
HPLC: High Performance Liquid Chromatography
UV: Ultraviolet
ELISA: Enzyme link immunosorbent assay
ICP-MS: Inductively coupled plasma mass spectrometry
Hs-CRP: High sensitivity C-reactive protein
LKR: Lankan rupee
PTH: Parathyroid hormone
RAE: Retinol activity equivalent
SSAO: Semicarbazide-sensitive amine oxidase
Cp: Ceruloplasmin
