# Supplementary material for: Micronutrient status and associated factors of adiposity in primary school children with normal and high body fat in Colombo municipal area, Sri Lanka
Source: BMC Pediatr. 2021 Jan 6;21:14. doi: 10.1186/s12887-020-02473-3 (PMC7786904; doi:10.1186/s12887-020-02473-3)
Supplement: Supplementary file 1 — Additional file 1: Table S1. Socio demographic, anthropometry and body composition characteristics of cases and controls (Sex specific and overall analysis). [file 12887_2020_2473_MOESM1_ESM.docx]

**Table S1** Socio demographic, anthropometry and body composition characteristics of cases and controls N=324

|  | Male | | | Female | | | Overall |  |  |
| --- | --- | --- | --- | --- | --- | --- | --- | --- | --- |
| Characteristics | Cases  % BF>28.6%  N=81 | Controls  ≤28.6%  N=80 | p  value | Cases  >33.7%  N=79 | Controls  ≤33.7%  N=84 | p value | Cases  N=160 | Control  N=164 | P value |
| Age in years | 9.11 ±0.323 | 9.21 ± 0.356 | 0.069^b*^ | 9.13 ±0.266 | 9.11 ± 0.292 | 0.572^b**^ | 9.12±0.29 | 9.16±0.32 | 0.306^b#^ |
| **Education level of father N (%)** |  |  |  |  |  |  |  |  |  |
| Completed primary education | 74 (91.4) | 56 (70.0) | 0.001^a*^ | 70 (88.6) | 74 (88.1) | 0.919^a**^ | 144 (90.0) | 130 (79.3) | 0.008^a#^ |
| Not completed | 7 ( 8.6) | 24 (30.0) |  | 9 (11.4) | 10 (11.9) |  | 16 (10.0) | 34 (20.7) |  |
| **Education level of mother N (%)** |  |  |  |  |  |  |  |  |  |
| Completed primary education | 75(92.6) | 59 (73.8) | 0.001^a*^ | 64 (81.0) | 74 (88.1) | 0.210^a**^ | 139 (86.9) | 133 (81.1) | 0.157^a#^ |
| Not completed | 6 (7.4) | 21 (26.2) |  | 15 (19.0) | 10 (11.9) |  | 21 (13.1) | 31 (18.9) |  |
| **Employment status of parents N (%)** |  |  |  |  |  |  |  |  |  |
| Both employed | 18 (22.2) | 8 (10.0) | 0.035^a*^ | 31 (39.2) | 21 (25.0) | 0.051^a**^ | 49 (30.6) | 29 (17.7) | 0.006^a#^ |
| One parent employed | 63 (77.8) | 72 (90.0) |  | 48 (60.8) | 63 (75.0) |  | 111(69.4) | 135(82.3) |  |
| **Monthly income ( USD) N (%)** |  |  |  |  |  |  |  |  |  |
| ≥411.06 USD | 46 (56.8) | 14 (17.5) | <0.001^a*^ | 27 (34.2) | 14 (16.7) | 0.01^a**^ | 73 (45.6) | 28 (17.1) | <0.001^a#^ |
| <411.06 USD | 35 (43.2) | 66 (82.5) |  | 52 (65.8) | 70 (83.3) |  | 87 (54.4) | 136 (82.9) |  |
| **Number of siblings N(%)** |  |  |  |  |  |  |  |  |  |
| ≤1 sibling | 36 (44.4) | 19 (23.8) | 0.006^a*^ | 33 (41.8) | 29 (34.5) | 0.341^a**^ | 69 (43.1) | 48 (29.3) | 0.009^a#^ |
| >1 siblings | 45 (55.6) | 61 (76.3) |  | 46 (58.2) | 55 (65.5) |  | 91 (56.9) | 116 (70.7) |  |
| Height (m) | 1.36 ±0.06 | 1.31 ±0.07 | <0.001^b*^ | 1.34± 0.05 | 1.29± 0.06 | <0.001^b**^ | 1.35±0.06 | 1.30±0.07 | <0.001^a#^ |
| Weight (Kg) | 39.46± 1.18 | 26.12±1.22 | <0.001^b*^ | 37.90± 1.16 | 25.54 ± 1.19 | <0.001^b**^ | 38.68±1.17 | 25.82±1.20 | <0.001^a#^ |
| BMI(kg/m^2^) | 20.80(19.1,23.2) | 14.64(13.8,17) | <0.001^c*^ | 21.02(19.2,22.3) | 14.96(13.7,16.3) | <0.001^c**^ | 21.0 (19.2, 22.8) | 14.89(13.7,16.6) | <0.001^c#^ |
| **BMI status N(%)** |  |  |  |  |  |  |  |  |  |
| Normal weight | 5 (6.2) | 70 (87.5) | <0.001^a*^ | 2 (2.5) | 78 (92.9) | <0.001^a**^ | 7 (4.4) | 148 (90.2) | <0.001^a#^ |
| Overweight | 51 (63.0) | 10 (12.5) |  | 57 (72.2) | 6 ( 7.1) |  | 108 (67.5) | 16 (9.8) |  |
| Obese | 25 (30.8) | 0 (00.0) |  | 20 (25.3) | 0 (00.0) |  | 45 (28.1) | 0 (0.0) |  |
| %Body fat | 35.8(31.5,40.2) | 16.26(12.6,24.1) | <0.001^c*^ | 38.4(35.5,42.2) | 20.7(15.5,27.1) | <0.001^c**^ | 37.68(33.8, 41.1) | 18.67(14.0,25.2) | <0.001^c#^ |
| Body fat (Kg) | 13.8(10.9,17.3) | 3.85(2.8,7.3) | <0.001^c*^ | 14.6(12.5,16.9) | 4.95(3.6,7.5) | <0.001^c**^ | 14.20(12.0, 17.1) | 4.55(3.2, 7.4) | <0.001^c#^ |
| WC (cm) | 71.09±1.08 | 55.37±1.11 | <0.001^b*^ | 72.59± 1.12 | 54.82±1.12 | <0.001^b**^ | 71.84±1.10 | 55.10±1.15 | <0.001^b#^ |
| WHtR | 0.53± 1.11 | 0.42± 1.09 | <0.001^b*^ | 0.53± 1.08 | 0.43± 1.09 | <0.001^b**^ | 0.53±1.10 | 0.42±1.09 | <0.001^b#^ |

BMI-Body mass index, WC-Waist circumference, WHtR- Waist- to- height Ratio BF-Body fat. LKR-Lankan rupee (1USD=133.80 LKR) *Differences between male cases and controls, **Differences between female cases and controls, ^#^ Differences between cases and controls (both males and females), ^a^Pearson chi-square value, ^b^independent sample, t-test- mean ± SD, ^c^Mann –Whitney U test with median (inter quartile range)-statistically significant at p<0.05.
