# Supplementary material for: Micronutrient status and associated factors of adiposity in primary school children with normal and high body fat in Colombo municipal area, Sri Lanka
Source: BMC Pediatr. 2021 Jan 6;21:14. doi: 10.1186/s12887-020-02473-3 (PMC7786904; doi:10.1186/s12887-020-02473-3)
Supplement: Supplementary file 2 — Additional file 2: Table S2. hs-CRP and leptin levels across the tertiles of micronutrients in the total study population. [file 12887_2020_2473_MOESM2_ESM.docx]

**Table S2**: hs-CRP and leptin levels across the tertiles of micronutrients in the total study population N=324

| Tertiles | hs-CRP [Median (IQR)] | Leptin [Median (IQR)] |
| --- | --- | --- |
| Vitamin A |  |  |
| T1 | 0.40 (0.12, 1.39) | 6.45 (2.12, 14.08) |
| T2 | 0.61 (0.10, 1.96) | 7.19 (3.77, 10.82) |
| T3 | 0.42 (0.12, 1.64) | 6.57 (2.57, 16.03) |
| p-value | 0.704^*^ | 0.409^*^ |
| Vitamin E |  |  |
| T1 | 0.37 (0.10, 1.46) | 7.05 (2.30, 14.44) |
| T2 | 0.32 (0.09, 1.46) | 5.34 (2.12, 12.79) |
| T3 | 0.87 (0.17, 1.84) | 7.83 (3.50, 14.86) |
| p-value | 0.083^*^ | 0.441^*^ |
| Vitamin D |  |  |
| T1 | 0.94 (0.18, 2.50) | 8.97 (3.99, 16.05) |
| T2 | 0.32 (0.09, 1.34) | 7.40 (2.89, 14.05) |
| T3 | 0.31 (0.09, 1.33) | 3.93 (2.01, 11.70) |
| p-value | **0.004*** | **0.001*** |
| Folate |  |  |
| T1 | 0.80 (0.17, 1.95) | 9.43 (4.31, 16.21) |
| T2 | 0.37 (0.12, 1.58) | 4.95 (2.48, 13.9) |
| T3 | 0.31 (0.09, 1.44) | 5.48 (1.79, 11.94) |
| p-value | **0.011*** | **0.007*** |
| RBC folate |  |  |
| T1 | 0.42 (0.10, 1.39) | 5.48 (1.98, 13.7) |
| T2 | 0.38 (0.09, 1.50) | 8.35 (3.17, 14.8) |
| T3 | 0.64 (0.16, 1.83) | 6.55 (2.88, 14.60) |
| p-value | 0.146* | **0.162*** |
| Ferritin |  |  |
| T1 | 0.14 (0.06,0.70) | 4.53 (1.79, 11.75) |
| T2 | 0.41 (0.13, 1.38) | 7.33 (2.41, 14.85) |
| T3 | 1.42 (0.43, 3.01) | 8.35 (3.80, 14.82) |
| p-value | **0.000*** | **0.008*** |
| Calcium |  |  |
| T1 | 0.31 (0.11, 1.22) | 5.13 (2.07, 12.23) |
| T2 | 0.69 (0.12,1.69) | 5.16 (2.18, 11.64) |
| T3 | 0.62 (0.09, 1.80) | 10.07 (4.83, 15.84) |
| p-value | 0.198^*^ | **0.001^*^** |
| Magnesium |  |  |
| T1 | 0.59 (0.18, 2.01) | 9.57 (3.78, 15.97) |
| T2 | 0.53 (0.11, 1.50) | 6.91 (3.17, 13.73) |
| T3 | 0.32 (0.07, 1.49) | 4.06 (1.88, 10.49) |
| p-value | **0.029^*^** | **0.000^*^** |
| Zinc |  |  |
| T1 | 0.40 (0.12, 2.06) | 6.58 (2.26, 16.10) |
| T2 | 0.53 (0.08, 1.77) | 7.82 (3.25, 14.01) |
| T3 | 0.62 (0.13, 1.53) | 5.82 (2.26, 11.77) |
| p-value | .0693^*^ | 0.606^*^ |
| Selenium |  |  |
| T1 | 0.32 (0.10, 1.26) | 6.46 (2.86, 16.90) |
| T2 | 0.71 (0.14, 1.85) | 7.05 (2.47, 13.96) |
| T3 | 0.55 (0.11, 1.43) | 6.41 (2.36, 11.96) |
| p-value | **0.539^*^** | **0.601^*^** |
| Copper |  |  |
| T1 | 0.17 (0.06, 0.64) | 4.75 (2.15, 14.37) |
| T2 | 0.44 (0.13, 1.77) | 5.33 (2.22, 10.09) |
| T3 | 1.43 (0.53, 2.66) | 9.77 (4.88, 15.92) |
| p-value | **0.000^*^** | **0.004^*^** |

**Table S2**: hs-CRP and leptin levels across the tertiles of micronutrients in the total study population N=324 *Continued*

| Tertiles | hs-CRP | Leptin |
| --- | --- | --- |
| Iron |  |  |
| T1 | 0.76 (0.25, 2.96) | 9.87 (4.71, 16.72) |
| T2 | 0.40 (0.12, 1.35) | 7.38 (2.86, 14.21) |
| T3 | 0.21 (0.08, 1.46) | 3.96 (1.80, 9.74) |
| p-value | **0.001^*^** | 0.**000^*^** |
| Chromium |  |  |
| T1 | 0.18 (0.08, 0.96) | 6.07 (2.05, 14.55) |
| T2 | 0.76 (0.19, 1.87) | 7.72 (2.68, 17.11) |
| T3 | 0.89 (0.15, 2.40) | 6.60 (3.14, 11.97) |
| p-value | 0.000^*^ | 0.449^*^ |
| Manganese |  |  |
| T1 | 0.51 (0.09, 1.47) | 6.46 (2.30, 12.46) |
| T2 | 0.64 (0.15, 1.99) | 6.64 (3.60, 14.85) |
| T3 | 0.41 (0.10, 1.58) | 7.21 (2.20, 13.75) |
| p-value | 0.884^*^ | 0.570^*^ |

hs-CRP- High sensitivity c- reactive protein,

Mann -Whitney U test with median (inter quartile range) -statistically significant at p<0.05, ^*^Differences between highest (T3) and lowest (T1) tertiles

T1, T2 and T3- Lowest, medium and highest tertiles.

The children with hs-CRP level >10mg/L excluded in ferritin and hs-CRP analysis
