# Supplementary material for: Micronutrient status and associated factors of adiposity in primary school children with normal and high body fat in Colombo municipal area, Sri Lanka
Source: BMC Pediatr. 2021 Jan 6;21:14. doi: 10.1186/s12887-020-02473-3 (PMC7786904; doi:10.1186/s12887-020-02473-3)
Supplement: Supplementary file 3 — Additional file 3: Supplementary file 1. Food Frequency Questionnaire (FFQ). This FFQ included the food and beverage items commonly consumed by Sri Lankan children. The traditional food items included in this questionnaire were pittu, string hoppers, hoppers, rotti, koththu (made out of rice/wheat flour), fermented food item (made out of black gram and rice), kesel muwa (banana flower), brinjal (eggplant), ambarella (edible fruit with fibrous pit used for cooking) and kola kanda (porridge made out of green leaves, rice and coconut milk) [file 12887_2020_2473_MOESM3_ESM.docx]

| Food type | Portion size | Rarely/Never  (only once or two times/month) | How many times per week | How many times per day |
| --- | --- | --- | --- | --- |
| **Rice & Rice flour products** |  |  |  |  |
| Rice (Red raw, White raw, Red Parboil, White parboiled, Samba, Basmati) |  |  |  |  |
| Fried rice |  |  |  |  |
| String hoppers(Red, White)/Noodles |  |  |  |  |
| Hoppers |  |  |  |  |
| Pittu |  |  |  |  |
| **Fermented food (Thosai, Idly)** |  |  |  |  |
| **Pulses** (Mung beans, Cowpea, Chickpea  Dhal, Black gram) |  |  |  |  |
| **Breakfast cereals** |  |  |  |  |
| Other |  |  |  |  |
| **Wheat flour products** |  |  |  |  |
| String hoppers |  |  |  |  |
| Pittu |  |  |  |  |
| Noodles |  |  |  |  |
| Rotti |  |  |  |  |
| Paratha / Koththu |  |  |  |  |
| Other (pancakes…) |  |  |  |  |

| Food type | Portion size | Rarely/Never  (only once or two times/month) | How many times per week | How many times per day |
| --- | --- | --- | --- | --- |
| **Bakery items/fast food** |  |  |  |  |
| Bread(White, Brown) |  |  |  |  |
| Pastries, rolls |  |  |  |  |
| Savory bun (Chicken, Fish, Vegetable, Seeni sambol, Egg, Burger) |  |  |  |  |
| Sweet bun |  |  |  |  |
| Pizza |  |  |  |  |
| Other |  |  |  |  |
| **Dairy products** |  |  |  |  |
| Cheese |  |  |  |  |
| Yoghurt |  |  |  |  |
| Curd with treacle |  |  |  |  |
| Ice cream |  |  |  |  |
| **Butter/Fat spread** |  |  |  |  |
| Milk |  |  |  |  |
| Other |  |  |  |  |
| **Vegetables** |  |  |  |  |
| Beans (Butter, wing, string beans) |  |  |  |  |
| Gourds (Bitter, Snake, Bottle, Labu ) |  |  |  |  |
| Kesel muwa / Ash plantain |  |  |  |  |
| Tomatoes |  |  |  |  |
| Drum stick, okra |  |  |  |  |
| Brinjal |  |  |  |  |
| Ambarella |  |  |  |  |
|  |  |  |  |  |
| Food type | Portion size | Rarely/Never  (only once or two times/month) | How many times per week | How many times per day |
| Carrot/ Pumpkin |  |  |  |  |
| Beetroot |  |  |  |  |
| Radish |  |  |  |  |
| Mushroom |  |  |  |  |
| **Leafy vegetables** |  |  |  |  |
| Green cabbage/cabbage/ Leeks |  |  |  |  |
| Dark green leaves, Spinach, Gotu kola/Sarana/Mukunuwanna |  |  |  |  |
| Other |  |  |  |  |
| **Fruits** |  |  |  |  |
| Citrus fruits |  |  |  |  |
| Fruits such as apple, guava, wood apple |  |  |  |  |
| Fruits such as banana, papaya, anoda |  |  |  |  |
| Avocado |  |  |  |  |
| Other |  |  |  |  |
| Tubers (Potatoes, sweet potatoes, manioc) |  |  |  |  |
| **Fish(White & Red)** |  |  |  |  |
| **Sprats** |  |  |  |  |
| **Dried fish** |  |  |  |  |
| **Sea food** |  |  |  |  |
| Prawns/ Crabs/Cuttle fish |  |  |  |  |
| **Meat (chicken, mutton, pork& beef)** |  |  |  |  |
| Processed meat |  |  |  |  |
| Organ meat(Liver, Gizzard, Brain) |  |  |  |  |
| Egg |  |  |  |  |
| Others |  |  |  |  |
|  |  |  |  |  |
| Food type | Portion size | Rarely/Never  (only once or two times/month | How many times per week | How many times per day |
| **Sweets & Snacks** |  |  |  |  |
| Chocolates/ Nutella |  |  |  |  |
| Candies/Toffees/ Jelly |  |  |  |  |
| Fruits with added sugar (fruit salad) |  |  |  |  |
| Biscuits (Sweet) |  |  |  |  |
| Biscuit (Savoury) |  |  |  |  |
| Cakes |  |  |  |  |
| Savory snacks (TipTip, potato chips, manioc chips, murukku, mixture) |  |  |  |  |
| Popcorn (Sweet, savory) |  |  |  |  |
| Pudding |  |  |  |  |
| Nuts (Peanuts, Cashew nuts) |  |  |  |  |
| French fries |  |  |  |  |
| **Drinks/Beverages** |  |  |  |  |
| Fresh fruit juice-Orange, Mango, Apple, Papaw |  |  |  |  |
| Flavored milk (Milo, Vanilla) |  |  |  |  |
| Fizzy drinks/Fruit flavoured juice (Packet) |  |  |  |  |
| Energy drink |  |  |  |  |
| Kola kanda |  |  |  |  |
| other |  |  |  |  |
|  |  |  |  |  |
|  |  |  |  |  |
|  |  |  |  |  |
|  |  |  |  |  |
