# Supplementary material for: Micronutrient status and associated factors of adiposity in primary school children with normal and high body fat in Colombo municipal area, Sri Lanka
Source: BMC Pediatr. 2021 Jan 6;21:14. doi: 10.1186/s12887-020-02473-3 (PMC7786904; doi:10.1186/s12887-020-02473-3)
Supplement: Supplementary file 4 — Additional file 4: Supplementary file 2. Physical activity questionnaire. This questionnaire was the adapted version of children physical activity questionnaire (C-PAQ) (https://www.mrc-epid.cam.ac.uk/wp-content/uploads/2014/08/CPAQ.pdf.) with culturally sensitive modifications where relevant. [file 12887_2020_2473_MOESM4_ESM.docx]

**MICRONUTRIENT STATUS, DIET, PHYSICAL ACTIVITY AND BODYCOMPOSITION IN PRIMARY SCHOOL CHILDREN IN THE COLOMBO MUNICIPAL AREA**

**CHIDREN'S PHYSICAL ACTIVITY QUESTIONNAIRE (C-PAQ)**

Parent Questionnaire

**ID**

Date …………………. Time started ………………………. Finished ………………………

Your child's name: ………………………………………………………………………………………………………..……

Your child's date of birth (dd/mm/yy): ………………../………………………/……………………….

Are you the child's: Mother / Father / Guardian / Other

Please note: - This questionnaire will take approximately 10 minutes to complete

- please answer the questions in relation to the child named above
- please complete every line in the questionnaire

**For further information, please contact:**

**Which of the following physical activities did your child do in the past 7 days?**

Please complete this questionnaire for the following days; ………………………………to ……………………………….

| Did you Child do the following activities in the past 7 days? | | **MONDAY – FRIDAY** | | **SATURDAY – SUNDAY** | |
| --- | --- | --- | --- | --- | --- |
|  | | How many times  Monday – Friday? | Total hours/minutes  Monday – Friday | How many times  Saturday – Sunday? | Total hours/minutes Saturday – Sunday? |
| **EXAMPLE :**  **Bike riding** | **No Yes** | **2** | **4 minutes** | **1** | **15 minutes** |
| SPORTS ACTIVITIES |  |  |  |  |  |
| Athletics | **No Yes** |  |  |  |  |
| Ellai/ Cricket | **No Yes** |  |  |  |  |
| Football/ Rugby/ Soccer | **No Yes** |  |  |  |  |
| Table Tennis/ /badminton/ Tennis squash | **No Yes** |  |  |  |  |
| Martial arts | **No Yes** |  |  |  |  |
| Netball/basket ball | **No Yes** |  |  |  |  |
| Dancing | **No Yes** |  |  |  |  |

| Did you Child do the following activities in the past 7 days? | | **MONDAY – FRIDAY** | | **SATURDAY – SUNDAY** | |
| --- | --- | --- | --- | --- | --- |
|  | | How many times  Monday – Friday? | Total hours/minutes  Monday – Friday | How many times  Saturday – Sunday? | Total hours/minutes Saturday – Sunday? |
| Running /Jogging | **No Yes** |  |  |  |  |
| Swimming | **No Yes** |  |  |  |  |
| Other (Please state) | **No Yes** |  |  |  |  |
| LEISURE TIME ACTIVITIES  Bicycle riding (not school travel) | **No Yes** |  |  |  |  |
| Household chores | **No Yes** |  |  |  |  |
| Play with pets | **No Yes** |  |  |  |  |
| Play in the park or garden | **No Yes** |  |  |  |  |
| Other (Please state) | **No Yes** |  |  |  |  |

| Did you Child do the following activities in the past 7 days? | | **MONDAY – FRIDAY** | | **SATURDAY – SUNDAY** | |
| --- | --- | --- | --- | --- | --- |
|  | | How many times  Monday – Friday? | Total hours/minutes  Monday – Friday | How many times  Saturday – Sunday? | Total hours/minutes Saturday – Sunday? |
| Hide and seek | **No Yes** |  |  |  |  |
| Climbing on trees | **No Yes** |  |  |  |  |
| Skipping rope | **No Yes** |  |  |  |  |
| Run and catch | **No Yes** |  |  |  |  |
| Walking alone/ with grandparents/Parents | **No Yes** |  |  |  |  |
| Walk for exercise | **No Yes** |  |  |  |  |
| Activities at School  Physical education class | **No Yes** |  |  |  |  |
| Travel by walking to school (to and from school = 2 times) | **No Yes** |  |  |  |  |
| other  please state | **No Yes** |  |  |  |  |

| Did you Child do the following activities in the past 7 days? | | **MONDAY – FRIDAY**  **Total hours/minutes** | **SATURDAY – SUNDAY**  **Total hours/minutes** |
| --- | --- | --- | --- |
|  | |  |  |
| **Example:**  **Watching TV/Videos** | **No Yes** | **15hrs** | **6hrs 30mins** |
| Art & craft (drawing, painting) | **No Yes** |  |  |
| Doing homework/Tuition class | **No Yes** |  |  |
| Imaginary play/ Play indoors with toys | **No Yes** |  |  |
| Listen to music | **No Yes** |  |  |
| Playing board games / cards | **No Yes** |  |  |
| Playing musical instrument | **No Yes** |  |  |
| Reading | **No Yes** |  |  |
| Sitting talking | **No Yes** |  |  |
| Travel by vehicle to school (to and from school) | **No Yes** |  |  |
| Travel by vehicle to any other places | **No Yes** |  |  |
| Screen hours (TV, videos/computer/Internet | **No Yes** |  |  |
| Other (please state): |  |  |  |
